# Te Pākeketanga: living and dying in advanced age - a study protocol

**DOI:** 10.1186/s12904-015-0073-4

**Published:** 2015-12-21

**Authors:** Merryn Gott, Tess Moeke-Maxwell, Lisa Williams, Stella Black, Gabriella Trussardi, Janine Wiles, Rangimarie Mules, Anna Rolleston, Ngaire Kerse

**Affiliations:** School of Nursing, University of Auckland, Boyle Building, 85 Park Road, Auckland, New Zealand; School of Population Health, University of Auckland, Auckland, New Zealand

**Keywords:** End of life, Older people, Advanced age, Indigenous people, Māori, Bereavement, Palliative care, Gerontology, Qualitative methods, Whānau caregiving, Family caregiving

## Abstract

**Background:**

The number of people dying in advanced old age is increasing rapidly and building the evidence base regarding end - of - life care for older people has been identified as an international policy priority. The unique opportunity to link longitudinal studies of ageing with studies exploring the end of life circumstances of older people remains under-exploited internationally. Very little is known about the specific circumstances, cultural needs and care preferences of indigenous older people, including Māori, at end - of - life and the needs of their whānau/ extended family carers.

**Methods:**

We will use rigorous qualitative methods to conduct post-bereavement interviews with bereaved whānau and family of 50–60 people who died >80 years; approximately half of participants will be Maori. The older decedents were participants in the first longitudinal study of older people involving a specific indigenous cohort internationally: *Te Puāwaitanga O Ngā Tapuwae Kia Ora Tonu, Life and Living in Advanced Age: a Cohort Study in New Zealand* (LiLACS NZ). Prior to death, they completed a questionnaire regarding their end-of-life preferences and nominated a family or whānau member to participate in this separate study exploring end-of-life circumstances of those in advanced age.

**Discussion:**

Recommendations to improve care will be formulated in collaboration with participants and their local hapū (sub-tribe). Ultimately this study has the potential to inform better outcomes for the growing numbers of people dying in advanced old age both in New Zealand and internationally, as well as their whānau and family caregivers. It also highlights the ability to generate an in-depth understanding of end-of-life circumstances by appending studies of palliative and end-of-life care onto existing longitudinal studies.

## Background

Research in palliative and end-of-life care faces unique challenges and there have been calls for the development of new methodological approaches to strengthen the evidence base [1]. One study design that does not appear to have been exploited to date is the appending of an end-of-life component onto longitudinal studies of ageing. Indeed, it has been almost 15 years since George argued that this method was a potentially ethically sensitive and cost-effective way to collect comprehensive information about end-of-life circumstances. However, to the best of our knowledge, it has not been adopted by large-scale longitudinal studies, despite their international proliferation in recent years [2].

One of the many benefits of this approach is the potential it brings to shed light on the end-of-life circumstances of older people, and in particular, the growing numbers of those dying in advanced age. The number of deaths worldwide will almost double in the next 50 years [3] and, in developed countries, will increasingly be concentrated in advanced age. In Aotearoa, New Zealand (NZ), the setting for the study reported in this paper, it has been estimated that 25 % of people dying with conditions known to benefit from a palliative care approach are aged over 85 years; [4] both this proportion, and the absolute numbers it represents, are increasing rapidly in line with an ageing population. The World Health Organisation has identified improving palliative and end-of -life care for older people as an international public health priority [5]. However, there is little evidence that the sheer scale of this challenge has been taken on board in research, practice, or policy either in New Zealand, or internationally.

Building the sparse evidence base to inform improvements in current care provision is vital for a number of reasons*.* Firstly, an international review concluded that many older people experience an end-of-life period marked by ‘repeated hospital admissions, under-recognition of symptoms, lack of preventive planning and inadequate home support’ [6]. A ‘huge inequality’ compared with younger age groups was identified in terms of access to publically funded palliative health services and a need for ‘considerable work’ was identified ‘to minimise the distress and maximise dignity for older people [and their families] at the end of their lives’ [6]. No research has been conducted regarding the situation in New Zealand. Secondly, specialist palliative care evolved to meet the needs of middle aged and ‘younger old’ people with cancer [7] and may not provide an appropriate framework for optimising end-of-life care in advanced age [6]. Finally, illness trajectories prior to death typically become more complex with increasing age and end-of-life experiences are also influenced by the wider socio-economic circumstances of later life; for some these include social isolation and economic hardship. International research confirms that these factors translate into high levels of (often unmet) physical and psychosocial need at the end-of-life [8, 9]. However, there has been very little integration between palliative care and gerontology at any level and an urgent need has been identified for research that situates dying within the wider context of ageing [10]. For all population groups, the relationship between ageing and dying remains under-theorised.

A further concern raised in relation to the discipline of palliative care as a whole has been the lack of attention paid to culture as a key determinant of preferences for, and experiences of, end-of-life. Indeed, it has been argued that paying more attention to culture would have benefits for the whole population: as Clark states, ‘putting culture at the centre of palliative care will be a key determinant of [future] efficacy and sustainability’ [11]. One aspect of culture that has received little attention within the literature is the needs of indigenous people at end-of-life. In New Zealand, the need to pay specific attention to the needs of Māori in all aspects of policy making is enshrined in Te Tiriti o Waitangi (the Treaty of Waitangi), the founding document of the nation. The limited research that has been done with Māori to date indicates a degree of dissonance between the nature of care and support preferred by Māori at the end-of-life and the current provision of specialist palliative care services [12]. This is unsurprising given that specialist palliative care is rooted in a Western philosophy and could be viewed within the New Zealand context as a colonial by-product. Furthermore, it is important to recognise that Māori may experience a number of additional barriers to achieving good end-of-life care, including poverty, racism, limited cultural competence amongst health professionals, and a lack of information about specialist palliative care amongst whānau and hapū (sub-tribal communities) and family [13, 14]. Consistent with a Māori understanding of ‘whānau’, the meaning of ‘family’ employed in the study includes extended family and friends who contribute a functionary role [15].

### Study design

#### Study aims and objectives

This study addresses the question: *What are the end-of-life circumstances of Māori and non-Māori dying >80 years and the experiences of their whānau and family during the end-of-life period and after bereavement?*

Adopting a particular focus on understanding the role of history, culture, and social circumstances in determining expectations and experiences from the bereaved whānau and family perspective, in this study we will:

1. Examine the experience of family and whānau end-of-life caregiving and bereavement, with a particular focus on barriers and facilitators to optimising their role in care provision;
2. Identify evidence of unmet physical and psychosocial needs prior to death, both amongst the deceased older person and their whānau and family carers;
3. Explore satisfaction with, and appropriateness of, publically funded service provision during the end-of-life period and into bereavement;
4. Determine the extent to which their deceased whānau and family member’s previously expressed priorities for care at the end-of-life were met;
5. Identify why priorities weren’t met if applicable;
6. Consider ways in which whānau and family members and their wider hapū (sub-tribal communities) and extended families can be better supported in their role of providing care and support for Māori and non-Māori dying in advanced old age.

### Study pilot

The final study design was determined by pilot work. This exploratory research involved conducting focus groups and interviews with 18 key stakeholders, including kuia (older Māori females), bereaved whānau, and care providers, and focused particularly upon the culturally specific requirements of exploring end-of-life issues with Māori. Participants reported several elements that were needed to ensure appropriate Māori participation and community support for the project: an articulation of the value of the research to local communities; the involvement of local interviewers in facilitating the research; data collection via face-to-face interviews within a supported environment; strong Māori representation within the research team; flexibility regarding the time between death and whānau interviews because of differential experiences of bereavement; the utility of whānau group interviews; and dissemination of findings to participants and communities through hui (meetings). These preliminary data informed our aims and objectives, as well as study design.

### The LiLACS NZ study

#### Overview

This paper presents the protocol for *Te Pākeketanga: Living and Dying in Advanced Age*. However it is also important to give sufficient detail regarding the longitudinal study of ageing to which it is linked, in order to place the study methods in context. *Te Puāwaitanga O Ngā Tapuwae Kia Ora Tonu, Life and Living in Advanced Age: a Cohort Study in New Zealand* (LiLACS NZ) inception cohorts were recruited in 2010 to examine predictors of successful advanced ageing amongst Māori and non-Māori, and is the first study of advanced ageing amongst an indigenous people to have been conducted internationally. Using Māori-centred methods, 421 Māori born between 1920 and 1930 (aged 80–90 years at project inception) were enrolled into the study, as well as 516 non-Māori born in 1925 (aged 85 years at project inception). Differential age cut-offs for Māori and non-Māori were adopted because of marked differences in life expectancy [16]. Participants were recruited within defined boundaries of the Bay of Plenty and Lakes District Health Boards (excluding Taupo region) NZ. Detailed information about health, service use, cultural, socio-economic, and whānau and family has been collected through interviewer administered questionnaires yearly: 2010 (W1), 2011 (W2) and 2012 (W3), 2013 (W4), 2014 (W5) and 2015 (W6) using mainly quantitative and clinical methods [17, 18]. NZ Health Information Service data (including hospitalisations) are also available. The study is rooted in the participants’ communities. Local organisations have been subcontracted to undertake data collection in seven research sites and provided with appropriate training from the LiLACS NZ investigators. The project is framed by the principles of Te Tiriti o Waitangi: protection, participation, and partnership. Māori are represented as investigators and Te Rōpū Kaitiaki o Ngā Tikanga Māori (Protectors of Principles of Conduct in Māori research), provide Māori direction for LiLACS NZ. Five waves of LiLACs NZ have been completed with a sixth Wave in progress. The full study protocol for LiLACS NZ has been published separately [17].

#### End-of-life questions

In Waves 3, 4 and 5, a questionnaire regarding preferences for end-of-life care was added to the interview covering the following areas: support/care priorities; preferences for place of end-of-life care/death; and wishes relating to funerals and tangihanga (traditional Māori funeral customs). This information was provided by 438 participants in wave 3 (147 Māori and 291 non- Māori) and 328 participants in Wave 4 (101 Māori and 227 non- Māori); wave 5 is still on-going. Participants were also invited to nominate, and consent to the project team contacting, a whānau/family member to be interviewed about their end-of-life care after their death. In addition, 76 % of Wave 3 and 4 participants nominated a whānau or family member to be interviewed after their death about the end-of-life care they received, providing a unique opportunity for a subsequent in-depth qualitative study to complement the quantitative methods of LiLACS NZ and explore end-of-life issues in detail. Those nominated were contacted at the time of nomination to gain verbal consent for future contact after the participant’s death.

The current LiLACS NZ cohort were born between 1920 and 1930. It is therefore unsurprising that a significant number of participants have already died. The death rate between Waves 1 and 2 was 9 % for Māori and 7 % for non-Māori; between Wave 2 and 3 it was 12 % for Māori and 7 % for non-Māori, between Wave 3 and 4 it was 11 % for Māori and 9 % for non-Māori, and between Waves 4 and 5 it was 15 % of Māori and 10 % for non-Māori.

### Te Paketaga: living and dying in advanced age

#### Study design and methods

##### Theoretical framework

As this is an area where little previous research has been conducted, a qualitative study design is most suitable to address the study aims. A social constructionist framework has informed our research aims and study design and will continue to frame all subsequent aspects of the project, including data collection and interpretation. Social constructivism understands reality as ‘relative to a specific conceptual scheme, theoretical framework, paradigm, form of life, society or culture’, and assumes there is a ‘non-reducible plurality of such conceptual schemes’ [19]. In other words, this approach emphasises the different ways in which people experience and understand events, an understanding which stands in contrast to the dominant positivist biomedical paradigm within which most palliative and end-of-life care research is conducted [20].

From a social constructionist approach, knowledge is viewed as co-constructed between participants and researchers. This understanding is congruent with the Health Promoting Palliative Care approach which also framed our study as it encourages the knowledge and expertise of whānau and family carers to be viewed as a valuable resource to be drawn upon. Health Promoting Palliative Care prioritises ‘the social context of beliefs and practices surrounding death, the tradition of the good death, the role of community’ and recognises ‘limits to health services contributions’ [21]. The process of socially constructing reality is understood as influenced by historical, cultural, economic and political factors [22]. This prompts us to pay specific attention not only to describing the circumstances of dying from our participants’ (multiple) perspectives, but also to endeavour to make visible the discursive processes and critical life events that shape their expressed understandings and experiences. This approach is congruent with our intention to contextualise end-of-life experiences for the deceased and bereaved within a broad socio-cultural, political and historical context; for Māori, we anticipate the impact of cultural disenfranchisement, urbanisation, and discrimination may be significant here [23]. Research conducted from a social constructionist perspective has already made a significant theoretical and practical contribution to other disciplines, for example gerontology [24, 25], but has not been an approach adopted within palliative and end-of-life care research which, overall, has been criticised for being atheoretical and not sensitive to context [26]. Adopting a social constructionist framework therefore not only provides the best ‘fit’ to our research aims, but will also enable the project to make an important contribution to the theoretical basis of the discipline of palliative care and promote closer, and much needed, integration with gerontology.

#### Rigour

To demonstrate rigour in our research we will draw upon the following ‘validation strategies’ for qualitative research: [27] 1) prolonged engagement in the field; 2) peer review and debriefing; 3) negative case analysis; 4) clarifying researcher bias; 5) member checking; and 6) rich, thick description which enables readers to make decisions about transferability. In line with an interpretive approach to qualitative research, we will also draw upon Angen’s (2000) framework [28] to ensure ‘trustworthiness’ which regards ‘validation’ as based on negotiation with participants and understands interpretations as temporal, located and open to reinterpretation. Her principles of ‘ethical validation’, i.e. the questioning of underlying moral assumptions, equitable treatment of diverse voices, and political and ethical implications of the research, and ‘substantive validation’, which requires research to provide some practical answers to questions, are also useful given the context of this project.

#### Ethical considerations

We have developed a protocol for post-bereavement interviewing which has now been used successfully in a number of projects, including in NZ and with Māori [29, 30]. This involves the ethical care processes embedded in Kaupapa Māori Research [31] which have been extended to both Māori and non- Māori. The LiLACS NZ community partner approaches the bereaved whānau and family (telephone, face-to-face, letter) to re-invite participation and to discuss the research aims and arrange interview details. The researchers make contact with participants prior to the interview to answer initial questions and enquire about catering needs as sharing kai (food) following a hui is customary. Two researchers attend each interview; both manaaki (care for) Te Pākeketanga participants to ensure the interview experience is as comfortable and as pleasant as possible for participants. The lead interviewer poses the research questions while the second researcher operates technical equipment and takes notes. Before the interview the researchers allow opportunity for whakawhanaungatanga (establishing connections), kai (sharing of food), karakia (prayer or blessing) if required by participants). Initial conversation prior to the interview ensures the nature of the research is understood. Consent forms are signed prior to digital recorders being turned on and a koha (gift of petrol voucher) is given prior to the interview commencing. In the majority of cases interviews are kanohi ki te kanohi (face to face) but two interviews were conducted via SKYPE and telephone. The interviewers check throughout the interview for participant distress, stop the interview at any time if this is desired and follow up with phone calls one week after the interview. If required, they refer the participant to their health professional of choice (with participant consent) on the very rare occasions this is desired by participants.

One ethical concern specific to this project is that we have collected information from the deceased LiLACS NZ participant about their end-of-life wishes. However, we do not have their consent to share this information with their whānau and family. A decision was made not to seek their consent for this given evidence that people can modify their responses to please or not upset family [32, 33]. Therefore, we will not share this information during interviews, nor will it be made available to the interviewers prior to the interview being conducted. Ethics approval for the study was granted by The University of Auckland Human Participants Ethics (reference number: 9686).

#### Recruitment

The LiLACS NZ team and local interviewers have developed strong links with potential participants, all of whom will have already received a card of condolence from the LiLACS NZ team following their bereavement. The first information about this study will be provided by a member of the local research team, who will verbally invite participation through face-to-face contact and provide details of the research team to contact if they are interested; the local interviewer will follow up the invitation with them and address any concerns (whilst being very clear they are under no obligation to participate). For both Māori and non-Māori participants, recruitment will be informed by the Pōwhiri Model of engagement (indigenous process of engaging with participants through collaborating with community partners and incorporating cultural customs) to ensure the rangatiratanga (respect for genealogical decent lines, personal status and cultural customs) of each whānau participant is cared for in a culturally meaningful knowledge exchange, based on the principles of manaakitanga (mutually beneficial collaboration between researchers and participants) [34]. The interviews will be conducted within four to six months following bereavement (avoiding events such as Christmas and providing flexibility to participants to decide when they are ready to participate). This is a timeframe similar to that adopted in previous research [35] including our own [36] and was recommended by participants in our pilot study; it represents a good balance between providing people with long enough to work through some of their initial grieving, but not so long that details of the circumstances of the death become forgotten.

#### Sample size

Our emphasis will be on providing a ‘thick description’ of the subject matter [37] which emphasises ‘sources of variation, nuances of difference, the elicitation of meaning and the understanding of processes, rather than the incidence and prevalence of particular circumstances and events’ [38]. In order to address the need to gather data to the same breadth and depth for the Māori and non-Māori population [39], approximately equal numbers of interviews will be conducted with each group. Our previous research experience, and the wider research literature, leads us to estimate that 25–30 interviews for Māori and 25–30 interviews for non-Māori will provide sufficient diversity of experience and enable detailed insight into the experiences of Māori and non- Māori participants. We therefore anticipate 50–60 interviews in total.

#### Interviews

All interviews will be conducted by TMM and SB (Māori) and LW and TMM (non-Māori) and will follow an in-depth ‘guided conversation’ format [40] which will enable participants to share the circumstances of their whānau or family member’s last months of life and their death, as well as their own caregiving and bereavement experiences prior to and following death. Interviews will be conducted at a place of the participants’ choosing, in their local community; our previous experience indicates they will likely last for between one and two hours. An interview guide was developed, informed by our pilot study, the literature, and our previous research. The guide can be administered in two ways depending on the interviewee’s preference and length of time available for the interview. The shorter interview involves the administration of a structured questionnaire that captures specific information about end-of-life circumstances. The Experiences at the End of Life questionnaire, consisting of 63 questions, was developed to capture data regarding the extent to which older LiLACS participants preferences for care – information collected as part of the longitudinal data collection in Waves 3, 4 and 5 – were met. It was reviewed for content and revised by Rōpū Kaitiaki of Ngā Tikanga Māori/Protectors of Principles of Conduct in Māori Research, to ensure appropriateness for Māori participants. The questionnaire consists of 63 items addressing the following domains:

- Te Pākeketanga participant personal details, including relationship to LiLACs NZ participant
- Whānau and LiLACs NZ participant’s experience of older age before death (living situation; health history; formal/informal care provided and interviewee's perceptions of whānau and family member’s satisfaction with life and involvement in care decisions)
- Satisfaction with health care in last 3 months of life of LiLACs NZ participant (overall)
- Satisfaction with health care in different settings (as applicable): public hospital; rest home/private hospital; statutory home care services; hospice facility
- After death circumstances
- Interviewee health questionnaire (physical/emotional state of health on day of interview).

The questionnaire will be administered in the portion of the interview exploring the end-of-life circumstances of the LiLACS NZ participants and additional qualitative data relating to this material will also be recorded.

The longer version of the interview schedule incorporates the Experiences at the End of Life questionnaire within a semi-structured interview format that also uses open-ended questions to gather in-depth contextual data. The schedule covers the following areas:

- Participant/carer personal details including relationship to whānau/family member
- Whānau/family member’s biographical details (early life: schooling, work, marriage, etc.)
- Whānau/family member’s experience of older age (living situation; health history; formal/informal care provided and interviewee's perceptions of whānau/family member’s satisfaction with life and involvement in care decisions)
- Whānau/family member’s end-of-life preferences including support and gaps in support (e.g. physical, psychological, social, and cultural needs; also whānau/family's support needs)
- Circumstances at end-of-life (using questionnaire items as discussion aide)
- Satisfaction with health care in last 3 months of life (overall)
- Satisfaction with health care in different settings (as applicable): public hospital; rest home/private hospital; statutory home care services; hospice facility
- After death circumstances and arrangements (funeral/tangihanga, who attended to the body, funeral planning, buried/cremation etc.)
- How interviewee is coping with bereavement
- Advice for other whānau /family caregivers
- Interviewee health questionnaire (physical/emotional state of health on day of interview).

Interviewing will be flexible and responsive to what participants’ share; the interview guide will also be modified throughout the research process to take into account new areas of relevance that are identified. For Māori participants interviews will be conducted in te reo Māori if requested and attention paid to gathering specific cultural knowledge about ageing and end-of-life reflecting knowledge held by whānau, hapū and iwi (tribal groups). Opportunity will also be provided for participants to discuss the extent to which services were culturally responsive to the needs of Māori. For all participants, end-of-life circumstances will be explored within the wider context of the whānau and family’s experience of the older person’s ageing. All interviews will be digitally recorded with permission and fully transcribed. Additional field notes will also be made immediately following the interview.

A summary report of each interview will be prepared by the research team. This not only functions as a ‘member checking’ activity but provides a taonga (treasured object) for families and whānau to keep. Whilst these reports will vary in length, in previous research they have typically been 15–20 pages long. This report would begin with an account of the deceased whānau and family member’s biography before laying out a condensed narrative of end-of-life circumstances, including funeral and tangihanga and the impact of death for whānau and family. Material will be informed by whānau and family narratives and set out under headings to facilitate reading, such as 'early life', 'adult years' 'older life' and 'end-of-life'. Whānau and family will have an opportunity to provide comment, amend, delete or change information; as such the summary report represents a collaboration between the researcher and the whānau. This approach was used very successfully by TMM in the Kia Ngawari project [41], where whānau identified that the reports made them feel as though they had been heard and had often become taonga (something treasured) that upholds the mana (prestige) of their loved one and the whānau and family as carer. The summary reports will also facilitate co-construction of Māori knowledge between researchers and whānau, reflecting Kaupapa Māori research which aims to ‘meet the expectations and quality standards set by Māori’ [39].

### Analysis

Transcribed interviews will be entered into NVivo 10, a software tool useful for managing large amounts of qualitative data. However, we are aware that NVivo can reduce whole stories to dismembered accounts [42] so analysis will always be mindful of the need to consider interview extracts within their appropriate context. A critical thematic analysis [43] will be applied to the data which, in the first instance, involves ‘initial’ line-by-line coding, an approach which helps ensure consideration of all ideas in the data. This process will also involve a ‘reflexive’ component (ibid) where researchers identify their existing preconceptions in relation to the data, helping them to avoid these subconsciously influencing their analysis and interpretation [44]. This approach is in line with Creswell and Miller’s validation strategy of ‘clarifying researcher bias’ [28]. Codes will then be developed into themes, and a central organising concept constructed which captures the essence of a theme. Following a review of individual themes, relationships between themes will be identified in order to develop theoretical insights grounded in the data.

Our analysis will also recognise not only what participants are saying, but how they are saying it; using narrative approaches to analysis in conjunction with other techniques works well to illuminate diverse experiences [44]. Participants’ narratives will also be examined as a whole to identify key points and explicit and implicit evaluations made by participants. In other words, we are interested in how participants talk about their experiences, as well as what they say. Such commentary by participants is revealing of social norms and values and acknowledging their narratives is also respectful of their own expertise and input. Māori analysis will be conducted by Māori researchers TMM and SB and informed by Māori cultural knowledge, customs and popular Māori health frameworks (e.g. Te Whare Tapa Whā). A separate Māori coding frame will be developed. Regular team workshops will also be held, separately for the Māori and non-Māori researchers in the first instance, where researchers code the same transcript as a means of enhancing the depth of the analysis and promoting rigour in the analysis. Negative case analysis, which involves identifying cases that deviate from the dominant pattern of a code, will also be undertaken to enhance rigour [28]. Once team members have reached consensus regarding emergent findings, these will be presented to participants through hui and group meetings, prior to finalising the study findings. These dissemination events will follow the model adopted successfully in the wider LiLACS NZ project and involve providing verbal feedback over a morning or afternoon session in two locations within each community – the marae and a community hall. It is important to note that, from a constructionist perspective, we do not view these events as a means to verify the ‘truth’ of the findings, but rather to establish their credibility with participants and further add to the richness of data interpretation and theoretical development by listening to participants views on our interpretation [28].

### Peer consultant focus groups

Following dissemination hui and group meetings with participants and final data analysis, findings will be fed back to local hapū in a series of ‘peer consultant focus groups’ with key stakeholders. This approach involves disseminating findings to ‘decision-makers’ and requesting their input to formulate recommendations which fit with on-going developments in other areas. Participants will comprise providers of publically funded, charitable and voluntary support services relevant to palliative and end-of-life care, as well as community leaders and kaumātua. We have used this technique successfully in previous research to identify workable and acceptable service improvements e.g. [45] and it is consistent with our overall approach which places participants and their communities at the heart of the research process.

## Discussion

This study represents an innovative approach to collecting in-depth data about the end-of-life circumstances of people in advanced age in the context of a longitudinal study. This is an area where very little is currently known, but new knowledge is urgently needed in light of projections that most future end-of-life care will need to be targeted to this age group, with whānau and family carers as key partners in care delivery. At an international level, in addition to a limited evidence base regarding end-of-life in advanced old age, insufficient attention has been paid to culture in all its manifestations as a determinant both of expectations and experiences of service users, and the way in which those services are organised and delivered. Further theoretical developments likely to arise from this project will help address the lack of theoretical integration between gerontology and palliative care which has been identified as hindering the development of integrated services in all countries, including NZ [46]. This information has the potential to make a significant contribution to improved outcomes for people in advanced old age at the end-of-life and their whānau and family.
